# A cryptic diagnosis: disseminated cryptococcal disease presenting as an asymptomatic pulmonary nodule and a skin lesion: a case report

**DOI:** 10.1186/1757-1626-1-430

**Published:** 2008-12-30

**Authors:** Ausami Abbas, Markus B Sikkel, Jonathan PW Collins, Chris WH Davies, Fabian Chen

**Affiliations:** 1 Department of Respiratory Medicine, Royal Berkshire Hospital NHS Trust, Reading, UK; 2 Department of HIV Medicine, Royal Berkshire Hospital NHS Trust, Reading, UK

## Abstract

**Background:**

Cryptococcosis refers to a spectrum of infections caused by the encapsulated yeast *Cryptococcus neoformans*. In the immunocompromised host cryptococcus may disseminate resulting in significant mortality and morbidity.

**Case presentation:**

We report the case of a 49-year-old homosexual male presenting with an atypical skin lesion associated with an asymptomatic pulmonary nodule. A subsequent diagnosis of disseminated cryptococcosis was made on India ink staining of cerebrospinal fluid.

**Conclusion:**

A diagnosis of cryptococcosis should be considered in all patients at risk of immunocompromise that present with asymptomatic pulmonary nodules in the presence of extrapulmonary manifestations, as exemplified by the unusual skin ulceration in our case.

## Case presentation

A previously well 49 year old homosexual man was referred to the on-call medical team with fever and a rash over his left loin. The patient believed that the rash had resulted from an insect bite which had occurred 9 weeks previously. It began as an erythematous patch but had since ulcerated, and was increasing in diameter despite 2 weeks of oral flucloxacillin and topical silver-nitrate dressings. The patient was previously fit and had no significant past medical history. He did not smoke and denied illicit drug use. No other risk factors for immunocompromise were identified.

**Figure 1:**
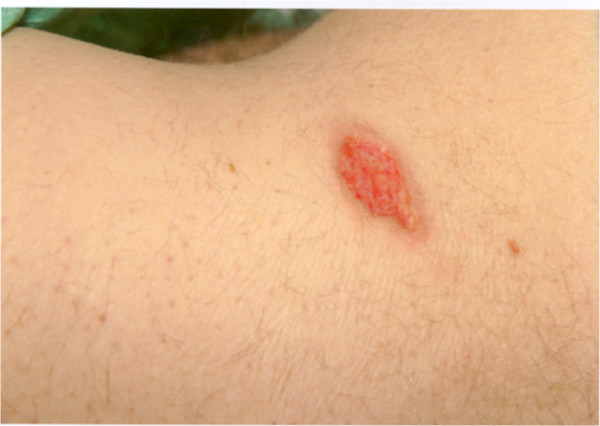
**Ulcerated lesion over his left loin**.

Examination revealed a 3 cm by 4 cm ulcerated lesion over his left loin. The base was a mixture of yellow slough and healthy-granulation tissue and the edges were well circumscribed (Fig 1). Further examination revealed a temperature of 39.1°C, dry mucous membranes and oral Candida. The remaining general and neurological examination was normal. The patient was admitted for intravenous fluid resuscitation and antibiotics.

**Figure 2:**
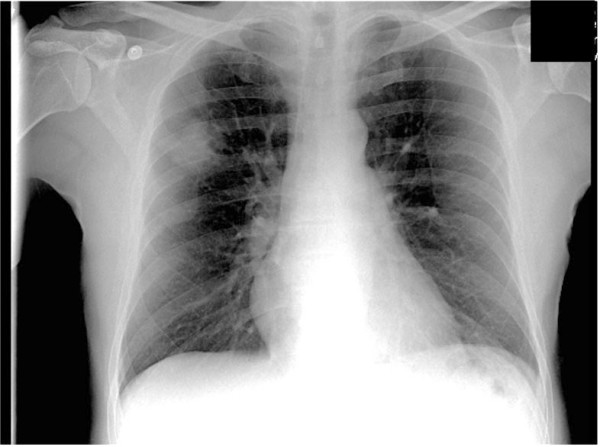
**Chest radiograph showing shadowing in the right upper zone**.

**Figure 3:**
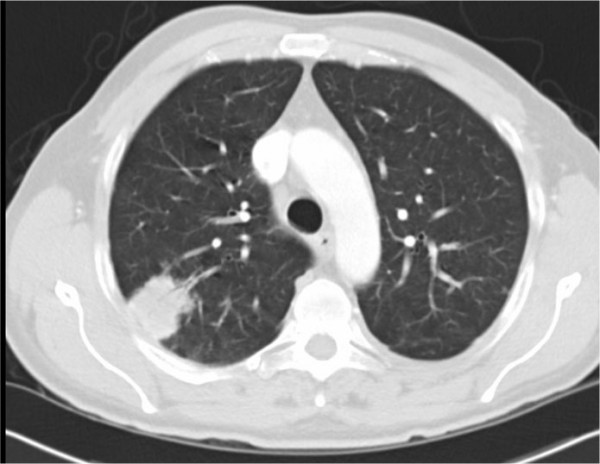
**Computed tomography (CT) imaging of the chest**.

Initial blood tests showed a mild microcytic anaemia (Hb 11.7 g/dL, MCV 76.0 fL) and lymphopaenia (WBC 2.78 × 10^9 ^L^-1^, Lymphocytes 0.44 × 10^9 ^L^-1^). HIV antibody titres at 3 days were positive. Chest radiograph on admission was abnormal; showing a 3.8 cm area of nodular shadowing in the right upper zone (Fig 2) which prompted computed tomography (CT) imaging of the chest (Fig 3).

The patient developed worsening headache associated with vomiting and mild altered mental status 4 days following admission. Urgent CT and Magnetic Resonance Imaging of the head showed mild cerebellar tonsil herniation through the foramen magnum but no contra-indication to lumbar puncture. This was performed and revealed an opening pressure of 33 cmH_2_O and cloudy cerebrospinal fluid. India ink staining and cryptococcal antigen testing of the cerebrospinal fluid was positive. This in conjunction with immunocompromise secondary to HIV confirmed the diagnosis of disseminated cryptococcosis.

The patient was treated with intravenous amphotericin B (1 mg/kg/day) plus flucytosine (100 mg/kg/day) for 2 weeks followed by fluconazole (400 mg/day) for 10 weeks. Daily therapeutic lumbar puncture was performed for 3 days until the opening pressure was less than 20 cmH_2_O. The patient was commenced on Highly Active Antiretroviral Therapy (HAART) and discharged with outpatient HIV specialist team follow up.

The patient remains well on HAART 12 months after initial presentation with no adverse neurological complications and no further episodes of opportunistic infections.

## Discussion

Cryptococcosis refers to a spectrum of infections caused by the encapsulated yeast *Cryptococcus neoformans*[1]. Immunosuppressed states associated with an increased risk of cryptococcal infection include HIV, organ transplantation, cancer (in particular haematological malignancies) and corticosteroid therapy[2].

Inhalation is the principal portal of entry for infection. Although pulmonary cryptococcosis usually presents as an asymptomatic pulmonary nodule, pneumonitis and acute respiratory distress syndrome can arise[3]. Pulmonary cryptococci may subsequently disseminate to involve other organs, most commonly the central nervous system causing meningioencephalopathy[3]. Cutaneous dissemination may occur, presenting as papules, pustules, plaques, ulcers or subcutaneous masses[4].

All patients with cryptococcal disease require lumbar puncture to exclude concomitant CNS involement[3]. Aggressive treatment of raised intracranial pressure (> 20 cm H_2_O) via daily serial therapeutic lumbar puncture is associated with a reduced overall mortality and morbidity[3].

## Conclusion

In our patient it is likely that the asymptomatic pulmonary cryptococcoma had disseminated as a result of immunocompromise secondary to HIV resulting in the cryptococcal skin ulcer and meningoencephalitis.

This case highlights the learning point that a diagnosis of cryptococcosis should be considered in all patients with potential immunocompromise presenting with asymptomatic pulmonary nodules. This is especially important in the presence of extrapulmonary manifestations, as exemplified by the unusual skin ulceration in our case.

## Consent

Written informed consent was obtained from the patient for publication of this case report and accompanying images. A copy of the written consent is available for review by the Editor-in-Chief of this journal.

## Competing interests

The authors declare that they have no competing interests.

## Authors' contributions

AA, JC, MBS, FC and CD were all involved in patient care. AA, JC and MBS were also involved in acquisition of data, analysis and interpretation of data, review of literature, drafting and revising the manuscript. FC and CD revised the manuscript for important intellectual content. All authors read and approved the final manuscript.
